# Circulating alpha-klotho levels are not disturbed in patients with type 2 diabetes with and without macrovascular disease in the absence of nephropathy

**DOI:** 10.1186/1475-2840-12-116

**Published:** 2013-08-14

**Authors:** Joris van Ark, Hans-Peter Hammes, Marcory C R F van Dijk, Marc G Vervloet, Bruce H R Wolffenbuttel, Harry van Goor, Jan-Luuk Hillebrands

**Affiliations:** 1 Department of Pathology & Medical Biology, Pathology, University of Groningen, University Medical Center Groningen, Hanzeplein 1, P.O. Box 30.001, Groningen, The Netherlands; 2 5th Medical Department, Section of Endocrinology, University Hospital Mannheim, University of Heidelberg, Mannheim, Germany; 3 Department of Pathology, Rijnstate Hospital, Arnhem, The Netherlands; 4 Department of Nephrology, VU University Medical Center, Amsterdam, The Netherlands; 5 Department of Endocrinology, University of Groningen, University Medical Center Groningen, Groningen, The Netherlands

**Keywords:** Atherosclerosis, Coronary artery disease, Klotho, Macrovascular disease, Peripheral artery disease, Type 2 diabetes

## Abstract

**Background:**

Diabetes is associated with a high incidence of macrovascular disease (MVD), including peripheral and coronary artery disease. Circulating soluble-Klotho (sKlotho) is produced in the kidney and is a putative anti-aging and vasculoprotective hormone. Reduced Klotho levels may therefore increase cardiovascular risk in diabetes. We investigated if sKlotho levels are decreased in type 2 diabetes and associate with MVD in the absence of diabetic nephropathy, and whether hyperglycemia affects renal Klotho production *in vitro* and *in vivo*.

**Methods:**

sKlotho levels were determined with ELISA in diabetic and non-diabetic patients with and without MVD, and healthy control subjects. Human renal tubular epithelial cells (TECs) were isolated and exposed to high glucose levels (15 and 30 mM) *in vitro* and Klotho levels were measured with qPCR and quantitative immunofluorescence. Klotho mRNA expression was quantified in kidneys obtained from long term (3 and 8 months) diabetic Ins2^Akita^ mice and normoglycemic control mice.

**Results:**

No significant differences in sKlotho levels were observed between diabetic patients with and without MVD (527 (433–704) pg/mL, n = 35), non-diabetic MVD patients (517 (349–571) pg/mL, n = 27), and healthy control subjects (435 (346–663) pg/mL, n = 15). High glucose (15 and 30 mM) did not alter Klotho expression in TECs. Long-term hyperglycemia in diabetic Ins2^Akita^ mice (characterized by increased HbA1c levels [12.9 ± 0.3% (3 months) and 11.3 ± 2.0% (8 months)], p < 0.05 *vs.* non-diabetic mice) did not affect renal *Klotho* mRNA expression.

**Conclusions:**

These data indicate that sKlotho levels are not affected in type 2 diabetes patients with and without MVD. Furthermore, hyperglycemia *per se* does not affect renal Klotho production. As type 2 diabetes does not alter sKlotho levels, sKlotho does not seem to play a major role in the pathogenesis of MVD in type 2 diabetes.

## Background

Patients with type 2 diabetes have a high incidence of macrovascular disease (MVD), including coronary artery disease (CAD) and peripheral artery disease (PAD) [1]. The transmembrane protein αKlotho (referred to as Klotho) is predominantly expressed in renal distal tubular epithelial cells where it functions as an obligate co-receptor with Fibroblast Growth Factor Receptor 1 (FGFR1) for Fibroblast Growth Factor 23 (FGF23), a phosphaturic hormone essential for maintaining mineral homeostasis [2]. In mice, Klotho deficiency is associated with accelerated and enhanced development of vasculopathy [3]. The extracellular domain of Klotho can be cleaved and shed in the circulation (*i.e.* soluble Klotho [sKlotho]) where it may function as a vasculoprotective hormone possibly by enhancing endothelial function [4,5] or direct inhibition of vascular calcification [6]. Recently, in agreement with this, higher sKlotho levels appeared to be independently associated with reduced prevalence of cardiovascular disease [7]. Moreover, in patients with chronic kidney disease (CKD) a graded reduction of urinary sKlotho has been described starting at an early stage of CKD, rendering sKlotho as a sensitive biomarker for early detection of CKD [6,8]. Finally, a reduction in renal *Klotho* gene expression has been observed in kidneys from patients with diabetic nephropathy [9].

Studies on sKlotho levels in diabetes are scarce and inconclusive and data have been obtained using various commercially available assays [10,11]. We need to interpret these data with caution, because a reliable ELISA-based assay to measure sKlotho levels has only recently become available [12]. Nevertheless, reduced sKlotho levels in type 2 diabetes could potentially be used as a biomarker for cardiovascular risk, and therefore studies on this are warranted. In addition, given its impact on both endothelial function and medial calcification, sKlotho may be involved in the pathogenesis of MVD in type 2 diabetes.

Against this background, in the current cross-sectional study we determined serum sKlotho levels in patients with type 2 diabetes with and without MVD, but without diabetic nephropathy. In addition we investigated the potential effect of hyperglycemia on renal Klotho expression. The following hypotheses were tested: 1) type 2 diabetes is associated with reduced sKlotho levels, particularly in patients with MVD and 2) hyperglycemia reduces renal Klotho expression. To this end, sKlotho ELISAs on patient sera were performed as well as *in vivo* mouse and *in vitro* cell culture experiments. Our data indicate that sKlotho levels are not affected in type 2 diabetic patients with and without MVD. This is supported by our *in vivo* and *in vitro* data showing that hyperglycemia *per se* does not affect renal Klotho production.

## Methods

### Study population

Patients with type 2 diabetes and non-diabetic subjects with and without MVD were included in this study. Individuals included are a subset of subjects on which we recently reported [13]. Patients were assigned to the following groups: diabetes, no MVD (n = 11); diabetes with CAD (n = 12); diabetes with PAD (n = 12); no diabetes with CAD (n = 13); and no diabetes with PAD (n = 14). Diagnosis of type 2 diabetes was based on criteria recommended by the WHO (http://whqlibdoc.who.int/publications/2006/9241594934_eng.pdf). In addition, age and sex-matched healthy control subjects (n = 15) were included in the study. Diagnosis of CAD was based on prior myocardial infarction (> 6 months), or of evidence of significant coronary artery stenosis during angiography. PAD was diagnosed based on a history of claudication or rest pain and assessed with bilateral peripheral arterial foot pulse examination and duplex ultrasonography. Patients with clinical evidence of both CAD and PAD were excluded from the study. Patients with clinically documented nephropathy (with eGFR < 60 mL/min/1.73 m^2^ or macroalbuminuria) were excluded from the study to exclude the potential confounding effect of kidney disease on sKlotho levels and presence of arterial disease. Additional exclusion criteria were: retinopathy, auto-immune diseases, neoplasms, acute or chronic infections, recent (< 6 months) surgery, age >80 yrs, hemodialysis and use of immunosuppressive agents. Participants were screened for cardiovascular risk factors including smoking, hypertension and BMI. In addition, laboratory measurements for glucose, HbA1c, lipid levels, blood urea nitrogen (BUN), serum creatinine, serum phosphate and serum calcium were performed. Estimated glomerular filtration rate (eGFR, mL/min per 1.73 m^2^) was calculated using the Chronic Kidney Disease Epidemiology Collaboration (CKD-EPI) equation [14]. Blood samples were obtained by venipuncture and collected in EDTA vacutainers for plasma isolation and in coagulation tubes for isolation of serum. Coagulation tubes were allowed to clot for a minimum of 30 minutes at room temperature before serum separation. Blood samples were centrifuged and plasma or serum was collected and stored at −80°C until further analysis. All participants gave written informed consent. The study protocol was approved by the local ethics committee of the University Medical Center Groningen (METc: 2008/335) and investigations were carried out in accordance with the principles of the Declaration of Helsinki.

### Spontaneous diabetic Ins2^Akita^ mice

Spontaneous diabetic heterozygous Ins2Akita^+/−^ (Ins2^Akita^) mice, purchased from Jackson Laboratory (Charles River Laboratories, Sulzfeld, Germany), were bred at the animal facility of the University Hospital Mannheim, University of Heidelberg. Age-matched non-diabetic homozygous Ins2Akita^−/−^ littermates served as control. Mice were housed in groups in cages with free access to standard food and water under a 12-h light and 12-h dark rhythm. Both male and female mice were used in this study. Glucose levels and body weight were monitored consecutively every other week, and HbA1c concentration was determined by affinity chromatography at the end of the study (MicromatII; Bio-Rad Laboratories, Munich, Germany). Insulin was occasionally given to individual diabetic mice to prevent critical weight loss. After 3 and 8 months of diabetes, kidneys were harvested under deep anesthesia and immediately frozen at −80°C until further analysis. Animal experiments were performed according to the ‘Principles of laboratory animal care’ (NIH publication no. 85–23, revised 1985; http://grants1.nih.gov/grants/olaw/references/phspol.htm) and were approved by the Institutional Animal Care and Use Committee of the University of Heidelberg.

### Klotho and cFGF23 ELISA

sKlotho levels were determined in human serum using a sandwich ELISA (Immuno-Biological Laboratories, Takasaki, Japan) with an intra-assay coefficient of variation (cv) and interassay cv of < 5% and < 8% respectively. c-Terminal FGF23 levels were determined in plasma using a sandwich ELISA (Immutopics, San Clemente CA) with an intra-assay cv of < 5% and interassay cv of < 16% [15]. Both assays were performed according to the manufacturer’s instructions. All samples and standards were measured in duplicate. Absorbance values were measured at 450 nm with a VarioSkan Microplate Reader (Thermo Scientific, Landsmeer, The Netherlands).

### Primary tubular epithelial cell (TEC) culture

TECs were isolated from a segment of healthy renal cortex, which was obtained from a nephrectomized kidney with renal cell carcinoma. The segment was taken distal from the tumor. The segment was cut into 1 mm^3^ pieces, washed in HBSS and subsequently placed in a T25 culture flask coated with FBS. The fragments were allowed to attach by putting the flask upside down for 1 hour at 37°C, 5% CO_2_. Next, 2.5 mL TEC medium (DMEM/Ham-F12 1:1 supplemented with Penicillin/Streptomycin, 25 mM HEPES, 2 mM L-glutamine, insulin-transferrin-sodium selenite supplement, hydrocortisone (36 ng/ml), epidermal growth-factor (10 ng/ml) and 5% heat-inactivated FBS) was gently added to the flask which was then put right-side-up in the incubator. Explant cultures were left untouched for 3 days after which 1 mL medium was added every 2–3 days. After 21 days, detached floating tissue fragments were removed and adherent TECs were detached using Trypsin/EDTA and seeded in new flasks (passage 1). Medium wash refreshed every 2–3 days.

### Immunostaining

Immunostaining was performed on TECs (passage 2) cultured in 8-well permanox chamber slides (Nalge Nunc, Creek Drive, USA). Subconfluent monolayers were washed with PBS and fixed for 15 minutes (room temperature) with 2% paraformaldehyde in PBS. After fixation, cells were washed twice with PBS and subsequently permeabilized in PBS/0.05% Triton for 3 minutes and washed with PBS. Next, cells were incubated with primary antibodies diluted in PBS/1% BSA for 60 minutes at room temperature. The following primary antibodies were used: mouse anti-cytokeratin (Clone AE1/AE3, DAKO, Glostrup, Denmark), mouse anti-cytokeratin 7 (Clone OV-TL 12/30, DAKO), mouse anti-epithelial membrane antigen (EMA, Clone E29, DAKO), and rat anti-Klotho (Clone KM2076, Transgenic Inc., Tokyo, Japan). For immunofluorescence, cells were subsequently incubated (30 minutes, room temperature) with species-specific AlexaFluor®555 conjugated-secondary antibodies (Life Technologies) diluted in PBS/1% BSA containing DAPI for nuclear counterstaining. Slides were mounted with Aqua PolyMount (Polysciences, Warrington, USA). Images were acquired with a Zeiss AxioObserver Z1 inverted microscope equipped with TissueFAXS acquisition software (Tissuegnostics, Vienna, Austria). For Klotho immunohistochemistry, cells were incubated for 30 minutes at room temperature with horseradish peroxidase (HRP)-conjugated rabbit anti-rat antibodies, which was followed by incubation with HRP-conjugated goat anti-rabbit antibodies for 30 minutes at room temperature. Peroxidase staining reaction was developed with 3-Amino-9-ethylcarbazole (AEC) as substrate. Nuclear counterstaining was performed with Mayer’s haematoxilin. Slides were mounted with Kaiser’s glycerin and images were aquired with an Olympus BX50 microscope.

### TEC stimulation and quantitative immunofluorescence

For *in vitro* stimulation experiments, TECs were seeded at a density of 2x10^4^ cells/cm^2^ in TEC medium containing 0.5% FBS and were allowed to attach for 24 hours. For hyperglycemic conditions, TECs were subsequently incubated for 96 hours in TEC medium containing 0.5% FBS and 7.8 mM (standard concentration), 15 mM or 30 mM D-glucose. Medium was refreshed every day. For stimulation with human serum, cells were seeded as described above. After 24 hours attachment the medium was replaced with TEC medium containing 5% 0.22 μm filtered human serum derived from 5 diabetic patients without MVD (average serum glucose level 6.5 mmol/L) or 5 healthy control subjects (average serum glucose level 5.2 mmol/L). TECs were incubated for 96 hours and medium was replaced every day. Next, cells were either fixed in 2% paraformaldehyde for quantitative immunofluorescence or lysed in RLT buffer (Qiagen, Venlo, the Netherlands) and stored in −80°C for subsequent RNA isolation.

To quantify Klotho protein expression *in vitro,* we performed Klotho immunofluorescence asdescribed above followed by quantitative TissueFAXS analysis. The total number of cells per mm^2^ surface area as well as the percentage Klotho^+^ cells and staining intensity under each experimental condition were determined using the TissueFAXS system (Tissuegnostics, Vienna, Austria), as described before [13]. To quantify the relative Klotho staining intensity, the mean fluorescence of intensity ratio (MFIR) was calculated by dividing the mean fluorescence intensity (MFI) of Klotho positive cells by the MFI of negative controls.

### Quantitative real-time PCR

Total RNA was isolated from mouse kidneys and human TECs using the RNeasy Micro kit (Qiagen) according to the manufacturer’s instructions. The RNA quantity was measured with a Nanodrop spectrophotometer. For cDNA synthesis, 1 ug RNA was converted to cDNA using SuperScript II reverse transcriptase and random hexamere primers (Life Technologies) according to the manufacturer’s instructions. *Klotho* mRNA expression was measured with a Taqman Gene expression assay (Applied Biosystems, Carlsbad, USA) using exon junction spanning primers for human *Klotho* (assay Hs00183100_m1) and mouse *Klotho* (assay Mm00502002_m1). For normalization of mouse *Klotho* expression, *B2M* (Mm00437762_m1) was included as a housekeeping gene. For normalization of human *Klotho* expression, *HPRT* was included as a housekeeping gene using in house developed primers (FW: GGCAGTATAATCCAAAGATGGTCAA; REV: GTCTGGCTTATATCCAACACTTCGT) and probe (CAAGCTTGCTGGTGAAAAGGACCCC) (Eurogentec Nederland B.V., Maastricht, The Netherlands). PCR reactions were performed on a LightCycler 480 II Real-Time PCR System (Roche Applied Sciences, Penzburg, Germany). Gene expression was analyzed with LightCycler 480 Software release 1.5.0 (Roche). To obtain the ΔCt, the Ct values of the respective housekeeping gene were subtracted from the *Klotho* Ct value. We used the comparative Ct method (2^-ΔCt^ method) to calculate relative *Klotho* gene expression.

### Statistical analysis

Normal distribution of continuous variables was tested with the Shapiro-Wilk test. For normally distributed data, the unpaired two-tailed Student’s *t*-test was used for comparisons between two groups and the one-way ANOVA with Bonferroni post-hoc was used for comparisons between three or more groups. For non-normally distributed data the non-parametric Mann–Whitney *U* test was used for comparisons between two groups. For comparisons between three or more groups of non-normally distributed data, the Kruskall-Wallis one-way analysis of variance was performed followed by pairwise comparisons using the Mann–Whitney *U* test with Bonferroni multiple testing correction. Dichotomous patient characteristics were compared with the *χ*2 test. Differences were considered significant at p < 0.05. Normally distributed data are reported as mean ± SEM, non-normally distributed data are reported as medians with interquartile range and categorical variables are presented as percentages. All data were analyzed using SPSS (version 20) and GraphPad Prism software (version 5).

## Results

### Patient characteristics

Characteristics of individuals included in this study are shown in Table 1. Diabetes duration of diabetic patients was 14.1 ± 0.9 years with HbA1c levels of 7.7 (6.8-8.5)% (60.1 (50.8-69.4) mmol/mol) (compared with 5.8 (5.5-6.0)% (39.3 (36.6-42.1) mmol/mol) in non-diabetic subjects, p < 0.01). No differences in glycemic control between subgroups of diabetic patients were observed (Table 1). There was no statistically significant difference in renal function between diabetic patients (sCr: 68 (81–80) μmol/L, eGFR: 89 (79–96) mL/min/1.73 m^2^) and non-diabetic subjects (sCr: 76 (64–83) μmol/L, eGFR: 94 (79–100) mL/min/1.73 m^2^) and no significant differences were observed between subgroups (Table 1). In line with this, BUN levels were similar among all groups. Additionally, no significant differences in serum phosphate and calcium levels were observed.

**Table 1:** Patient characteristics

	**Diabetes**				**No Diabetes**			
	**No MVD (n = 11)**	**CAD (n = 12)**	**PAD (n = 12)**		**Healthy control (n = 15)**	**CAD (n = 13)**	**PAD (n = 14)**	**p value**
**Demographics**								
Age (years)	56.0 (50.0-63.0)^a^	65.5 (62.0-71.0)	70.5 ± (63.5-74.0)		55.0 (53.0-56.0)^b^	57.0 (53.0-71.0)	59.5 (53.0-64.0)	p < 0.05
Sex (% male)	5 (46)	6 (50)	6 (50)		8 (53)	9 (69)	8 (57)	NS
Body mass index (kg/m2)	32.6 ± 2.6^c^	32.2 ± 2.1^c^	28.5 ± 3.1		24.5 ± 0.8	27.1 ± 1.0	23.6 ± 0.9	p < 0.001
Diabetes duration (years)	14.2 ± 1.0	16.8 ± 1.5^d^	11.3 ± 1.6		-	-	-	p < 0.05
Smoking (%)	3 (27)	1 (8)	2 (17)		2 (13)	5 (39)	8 (57)^i^	p < 0.05
**Metabolic variables**								
WBC count (10^6^/mL)	7.9 (5.7-11.9)	7.0 (5.8-7.4)	8.7 (7.4-10.2)		5.6 (4.4-6.4)^e^	5.5 (5.1-6.8)	8.1 (5.9-9.2)	p < 0.05
Glucose (mmol/L)	7.1 ± 0.4^f^	9.7 ± 1.5^f^	9.5 ± 1.5^f^		5.3 ± 0.1	-	6.0 ± 0.6	p < 0.001
HbA1c - (%)	7.9 (7.2-9.8)^f^	8.3 (6.8-8.5)^f^	7.4 (6.6-8.4)^f^		5.6 (5.5-5.9)	5.8 (5.6-6.0)	5.6 (5.5-6.0)	p < 0.001
- (mmol/mol)	62.8 (54.6-83.1)^f^	66.7 (50.8-69.4)^f^	57.4 (48.6-68.3)^f^		37.7 (36.6-41.0)	39.9 (37.7-42.1)	37.7 (36.1-42.1)	p < 0.001
Cholesterol (mmol/L)	4.2 (3.6-4.7)	4.3 (3.8-5.2)	4.3 (3.8-4.5)		5.8 (5.0-6.3)^g^	4.0 (3.6-4.5)	4.4 (3.4-5.9)	p < 0.01
Triglyceriden (mmol/L)	1.6 (1.1-2.1)	2.1 (1.7-3.0)	1.9 (1.3-2.2)		1.5 (1.0-1.8)	1.4 (0.9-2.1)	1.9 (0.9-2.8)	NS
HDL-cholesterol (mmol/L)	1.3 (1.2-1.4)	1.1 (1.1-1.3)^h^	1.1 (0.9-1.4)		1.5 (1.4-1.9)	1.2 (1.2-1.5)	1.3 (1.0-1.7)	p < 0.05
LDL-cholesterol (mmol/L)	2.3 (1.9-2.6)	2.1 (2.0-3.1)	2.1 (1.6-2.7)		3.6 (3.3-4.0)^g^	2.2 (1.9-2.8)	2.3 (1.7-3.5)	p < 0.01
Creatinine (μmol/L)	64.5 (53.5-76.0)	68.5 (66.0-79.0)	68.0 (57.0-89.0)		75.0 (63.0-82.0)	80.0 (75.0-84.0)	71.0 (63.0-79.0)	NS
eGFR (mL/min/1.73 m^2^)	97.3 (87.0-107.4)	86.1 (82.7-90.3)	80.8 (73.2-90.4)		94.9 (87.1-97.9)	90.0 (66.6-99.1)	99.7 (80.2-101.5)	NS
BUN (mmol/L)	6.2 (4.7-7.0)	6.5 (5.5-7.4)	7.0 (5.9-7.9)		6.2 (5.5-7.0)	6.3 (5.9-6.9)	6.2 (4.4-6.5)	NS
Phosphate (mmol/L)	1.1 ± 0.1	1.2 ± 0.1	1.4 ± 0.1		1.2 ± 0.1	1.3 ± 0.1	1.4 ± 0.1	NS
Calcium (mmol/L)	2.7 (2.5-2.8)	2.9 (2.5-3.4)	2.9 (2.4-3.2)		2.7 (2.3-2.9)	2.9 (2.6-3.1)	2.9 (2.5-3.3)	NS
sKlotho (pg/mL)	581.4 (496.8-651.9)	561.0 (463.2-747.0)	493.7 (287.8-777.7)		434.7 (345.8-662.9)	490.5 (349.0-549.4)	518.9 (401.5-570.5)	NS
FGF23 (RU/mL)	131.0 (118.5-147.0)	117.5 (106.0-256.0)	149.0 (120.0-237.0)		99.5 (87.0-119.0)	97.0 (88.0-112.0)	121.0 (105.0-140.0)	NS
**Medication**								
Insulin (%)	10 (91)	11 (92)	8 (67)		-	-	-	NS
Oral glucose-lowering	6 (55)	8 (67)	10 (83)		-	-	-	NS
agents (%)								
Statins (%)	7 (64)	11 (92)	10 (83)		1 (7)^i^	13 (100^)^i	10 (71)	p < 0.001
ACE inhibitors (%)	7 (64)	5 (42)	7 (58)		1 (7)^i^	10 (77)^i^	6 (43)	p < 0.01
Angiotensin II inhibitor (%)	2 (18)	4 (33)	1 (8)		0 (0)	1 (8)	2 (14)	NS
Alpha blockers (%)	1 (9)	0 (0)	0 (0)		0 (0)	0 (0)	0 (0)	NS
Beta blockers (%)	1 (9)	7 (58)	3 (25)		1 (7)^i^	12 (92)^i^	3 (21)	p < 0.001
Calcium antagonists (%)	4 (36)	7 (58)^i^	5 (42)		0 (0)^i^	1 (8)	3 (21)	p < 0.01
Diuretics (%)	6 (55)	9 (75)^i^	6 (50)		1 (7)^i^	4 (31)	3 (21)	p < 0.01
Antiaggregants (%)	2 (18)^i^	9 (75)	7 (58)		0 (0)^i^	12 (92)^i^	11 (79)^i^	p < 0.001
Anticoagulants (%)	1 (9)	0 (0)	1 (8)		0 (0)	2 (15)	1 (7)	NS

Data are presented as percentage, or as mean ± SEM for normally distributed data, or as median (interquartile range) for skewed data as appropriate. Statistically significant with ANOVA compared with: ^a^diabetes with PAD. ^b^diabetes with CAD, diabetes with PAD. ^c^no diabetes with PAD, healthy control. ^d^diabetes with PAD. ^e^diabetes without MVD, diabetes with PAD, no diabetes with PAD. ^f^all groups without diabetes. ^g^diabetes without MVD, diabetes with PAD, no diabetes with CAD. ^h^healthy control. ^i^statistically significant with *χ*2 test. NS: not significant.

### Serum sKlotho and plasma cFGF23 levels

**Figure 1:**
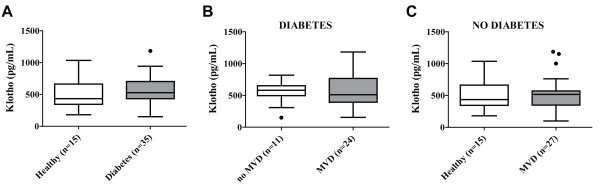
**Klotho serum levels are not reduced in type 2 diabetes and MVD. (A)** No differences in serum sKlotho levels are present between healthy control subjects and type 2 diabetes patients with and without MVD. **(B)** Within type 2 diabetes patients, no difference in sKlotho levels between patients with or without MVD is present. **(C)** Similarly, when comparing non-diabetic patients with MVD with healthy control subjects no difference in sKlotho concentration was detected. Data are expressed as Tukey’s box-and-whisker plots.

Serum sKlotho concentration was measured with ELISA (Table 1). No difference in sKlotho levels between healthy control subjects and diabetic patients was observed (Figure 1A). Also, no difference in serum sKlotho levels between diabetic patients with and without MVD was detected (Figure 1B). Similarly, MVD in non-diabetic subjects was not associated with reduced serum sKlotho levels either (Figure 1C). Furthermore, sKlotho levels were not significantly associated with serum creatinine levels or eGFR in diabetic and non-diabetic subjects. Reduced renal Klotho expression (independent of sKlotho levels) might be associated with a compensatory increase in circulating FGF23, due to FGF23 resistance. However, plasma cFGF23 levels did not significantly differ between diabetic patients and non-diabetic subjects and no differences were observed among the different subgroups (Table 1). Furthermore, use of any medication was not associated with differences in sKlotho or FGF23 levels.

### TEC phenotype

**Figure 2:**
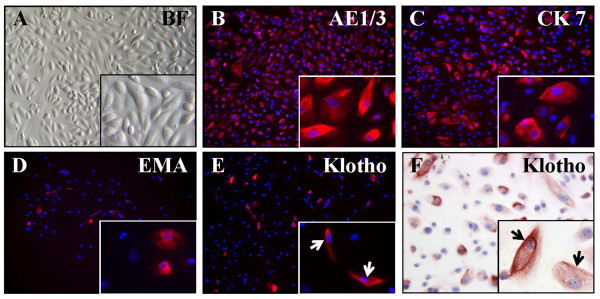
**TECs isolated from kidney explants express renal tubular markers and produce Klotho. (A)** Cultured TECs form a monolayer containing cells with heterogeneous morphology. Immunofluorescence revealed extensive immunoreactivity with antibodies against epithelial markers Pan-cytokeratin (AE1/3) **(B)**, Cytokeratin 7 (CK7) **(C)** and Epithelial Membrane Antigen (EMA) **(D)**. Moreover, TECs produce Klotho as shown with immunofluorescence **(E)** and immunohistochemistry **(F)**. Nuclear staining is shown in blue while positive staining with antibodies is shown in red (AlexaFluor®555 for immunofluorescence and AEC for immunohistochemistry). Arrows indicate Klotho positive epithelial cells. BF: Brightfield. Magnification: 200x; magnification, insets: 630x.

For *in vitro* studies on renal Klotho production primary TECs were isolated, expanded and phenotyped. TECs consisted of a morphologically heterogeneous cell population consisting of cobblestone and elongated spindle-shaped cells (Figure 2A). Most cells showed positive staining with antibodies against pan-cytokeratin marker AE1/3, indicating that TECs belong to the epithelial lineage (Figure 2B). A smaller proportion of TECs expressed the distal tubule markers cytokeratin 7 (Figure 2C) and EMA (Figure 2D). TECs thus represent a mixture of different renal tubular epithelial cell types. Strong positive im-munostaining for Klotho was observed in a minority of cells (Figure 2E,F)*.*

### Effect of hyperglycemia and type 2 diabetic patient-derived serum on renal Klotho production

**Figure 3:**
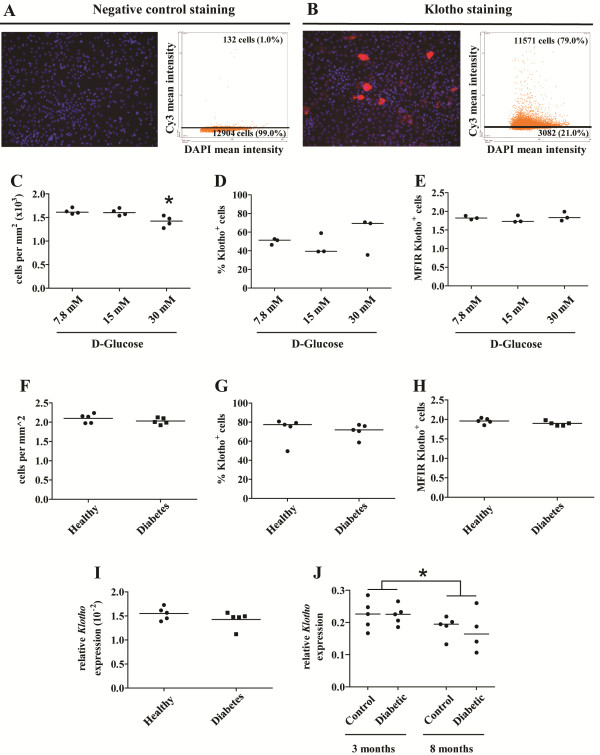
**Hyperglycemia and diabetes patient-derived serum do not alter Klotho production in TECs *in vitro*. (A**, **B)** Representative immunofluorescence images of TECs stained for Klotho and corresponding scatterplots produced by quantitative TissueFAXS analysis. **(A)** Negative control staining (primary antibody omitted) with corresponding scatterplot. **(B)** Immunofluorescence image of TECs cultured in the presence of 30 mM glucose, stained for Klotho, with corresponding scatterplot. **(C)** The total number of TECs *in vitro* was significantly lower when cells were incubated with medium containing 30 mM glucose compared with lower glucose concentrations. However, there was no effect of hyperglycemic culture conditions on the percentage of Klotho^+^ cells **(D)** or the Klotho staining intensity **(E)**. In addition, serum derived from diabetic subjects did not affect the total number of cells **(F)**, percentage of Klotho^+^ cells **(G)**, the Klotho staining intensity **(H)** or the relative level of *Klotho* mRNA expression **(I)** compared with serum derived from healthy control subjects. **(J)** No difference in renal *Klotho* mRNA expression was observed between hyperglycemic Ins2^Akita^ and normoglycemic control mice. However, a significant reduction in renal *Klotho* mRNA expression was detected in 8 months old mice compared with 3 months old mice, independent of the presence of diabetes. Data are expressed as scatterplots with the horizontal bar representing the median; *p < 0.05.

Next, we analyzed whether hyperglycemia affects renal Klotho production *in vitro*. Additionally, to investigate if other circulating factors in diabetic patients influence renal Klotho production, TECs were cultured in the presence of serum from diabetic patients or healthy control subjects. Quantitative immunofluorescence was used to quantify the numbers of Klotho-expressing cells as well as the expression level (Figure 3A,B). Exposure to 30 mM glucose decreased the total number of TECs (Figure 3C). However, the percentage of Klotho^+^ cells (Figure 3D) and Klotho staining intensity of Klotho^+^ cells (Figure 3E) was not altered after exposure to high glucose. Quantitative real-time PCR confirmed the expression of *Klotho* mRNA in TECs. However, *Klotho* mRNA expression was not altered after exposure to high glucose (data not shown). These data suggest that hyperglycemia does not influence renal Klotho production. Similar results with regard to total cell number (Figure 3F), percentage Klotho^+^ cells (Figure 3G), Klotho staining intensity (Figure 3H) or *Klotho* gene expression (Figure 3I) were obtained after culture of TECs in the presence of serum derived from diabetic patients.

### Renal Klotho mRNA expression in diabetic Ins2^Akita^ mice

To study whether hyperglycemia affects renal Klotho expression *in vivo*, kidneys from 3 and 8 months hyperglycemic Ins2^Akita^ mice were analyzed. Diabetic Ins2^Akita^ mice had significantly increased HbA1c levels at both 3 months (13.1 (12.6-13.3)% (119.7 (114.2-121.9) mmol/mol) *vs.* 6.2 (6.0-6.6)% (44.3 (42.1-48.6) mmol/mol), p < 0.0001) and 8 months (10.9 (7.9-14.7)% (95.6 (62.3-137.2) mmol/mol) *vs.* 6.5 (5.4-6.6)% (47.5 (35.5-48.6) mmol/mol), p < 0.05) when compared with age-matched wild-type control mice, confirming a severe hyperglycemic state in diabetic Ins2^Akita^ mice. No difference in renal *Klotho* gene expression in 3 and 8 months hyperglycemic Ins2^Akita^ mice was observed when compared with age-matched normoglycemic control mice (Figure 3J). Ageing was clearly associated with significantly reduced *Klotho* gene expression (Figure 3J).

## Discussion

This study demonstrates that circulating sKlotho levels are similar in patients with type 2 diabetes and healthy individuals in the absence of nephropathy. In addition, no difference in sKlotho levels was observed between MVD patients and healthy control subjects, independent of the presence of diabetes. The lack of a decline in sKlotho in diabetic subjects with or without MVD can have several explanations. First, the null hypothesis might be true, implying that changes in sKlotho levels are not involved in the pathogenesis in MVD, independent from age or kidney function. Moreover, the widespread use of ACE-inhibitors and angiotensin receptor blockers in the diabetic subjects may have counterbalanced a presumed negative effect of diabetes on Klotho production [16]. Finally, it has been shown that insulin promotes cleavage of Klotho from the plasma membrane by ADAM10 and ADAM17, generating sKlotho [17]. Therefore, exogenously administered insulin in diabetic patients might compensate diabetes induced sKlotho depletion by increasing sKlotho shedding from the cell membrane. Our *in vitro* data are however not in favor of these explanations, but rather strengthen our conclusion that diabetes does not influence renal Klotho expression.

Our data are in contrast with results reported by Semba *et al.* showing that the presence of CVD is independently associated with reduced sKlotho levels [7]. A potential explanation for this discrepancy might be that in the present study only patients with isolated CAD or PAD were included, while in the study by Semba *et al.* CVD patients with stroke and heart failure were included as well. Similar to our data, in the study by Semba *et al.* neither CAD nor PAD were separately associated with lower sKlotho levels.

Dysregulation of the FGF23-Klotho axis contributes to disturbed mineral homeostasis and as such might increase cardiovascular risk in diabetes. Therefore, we additionally measured cFGF23 levels in our cohort. However, like sKlotho, no differences in cFGF23 levels among the different study groups were observed. In line with this, serum calcium and phosphate levels were similar among all groups.

Circulating sKlotho concentration may not necessarily reflect total renal Klotho production, and despite similar sKlotho levels in diabetic and non-diabetic individuals, membrane-bound renal Klotho expression might be affected as a consequence of diabetes. Studies on this would have required kidney biopsy in our cohort. As a surrogate we performed *in vitro* experiments with TECs exposed to high glucose. In addition, we measured renal *Klotho* gene expression in long-term hyperglycemic Ins2^Akita^ mice. Both the *in vitro* and *in vivo* mouse experiments revealed that renal Klotho production is not affected by hyperglycemia per se. Furthermore, the *in vitro* data suggest that circulating factors other than glucose in type 2 diabetes (such as glycated proteins or proinflammatory cytokines) do not influence renal Klotho production, as no difference in Klotho production between TECs exposed to serum derived from diabetic patients and healthy control subjects was observed.

Interpretation of results from different studies on sKlotho is still complicated by the fact that different assays (which are not all reliable and validated) are being used by different investigators. The results described here are in line with a recent study by Seiler *et al.* using the same Klotho assay, which showed that sKlotho levels are in fact not associated with deteriorated kidney function in CKD patients, and have poor predictive value for all-cause mortality after 2-years follow up. Furthermore, low sKlotho serum levels were not associated with an increased prevalence of diabetes in this study [18].

## Limitations

The present study is somewhat limited by a small sample size per group which may have obscured small differences in sKlotho levels between groups. Our *in vitro* studies were limited by the measurement of total cellular Klotho mRNA and protein under normo-and hyperglycemic culture conditions. We cannot exclude that the cleavage and excretion of sKlotho is affected by increased glucose levels *in vitro*.

## Conclusions

Despite the recent interest in sKlotho as a biomarker for renal and cardiovascular disease, our study did not provide a rationale for using sKlotho levels as biomarker for MVD in type 2 diabetic patients and non-diabetic subjects in the absence of nephropathy. Based on our data, a major role of sKlotho in the pathophysiology of MVD in diabetes is unlikely.

## Abbreviations

ADAM: A Disintegrin and metalloproteinase domain-containing protein; BUN: Blood urea nitrogen; CAD: Coronary artery disease; CKD: Chronic kidney disease; CVD: Cardiovascular disease; eGFR: Estimated glomerular filtration rate; EMA: Epithelial membrane antigen; FGF23: Fibroblast growth factor 23; FGFR1: Fibroblast growth factor receptor 1; MVD: Macrovascular disease; PAD: Peripheral artery disease; sKlotho: Soluble Klotho; TEC: Tubular epithelial cell; WBC: White blood cells.

## Competing interests

The authors declare that they have no competing interests.

## Authors’ contributions

JvA was responsible for the conception and design of the study, researched data, and wrote the manuscript. HPH contributed to acquisition of data, discussion and reviewed/edited manuscript. MCRFvD contributed to acquisition of data and reviewed/edited manuscript. MGV contributed to acquisition of data, discussion and reviewed/edited manuscript. BHRW coordinated inclusion of diabetic individuals and reviewed/edited the manuscript. HvG contributed to discussion and reviewed/edited manuscript. JLH was responsible for the conception and design of the study, researched data, contributed to discussion and reviewed/edited manuscript. All authors read and approved the final manuscript.
