# Correction: Circulating alpha-klotho levels are not disturbed in patients with type 2 diabetes with and without macrovascular disease in the absence of nephropathy

**DOI:** 10.1186/1475-2840-12-120

**Published:** 2013-08-28

**Authors:** Joris van Ark, Hans-Peter Hammes, Marcory CRF van Dijk, Chris PH Lexis, Iwan CC van der Horst, Clark J Zeebregts, Marc G Vervloet, Bruce HR Wolffenbuttel, Harry van Goor, Jan-Luuk Hillebrands

**Affiliations:** 1 Department of Pathology & Medical Biology, Pathology, University of Groningen, University Medical Center Groningen, Hanzeplein 1, PO Box 30.001, Groningen, The Netherlands; 2 5th Medical Department, Section of Endocrinology, University Hospital Mannheim, University of Heidelberg, Mannheim, Germany; 3 Department of Pathology, Rijnstate Hospital, Arnhem, The Netherlands; 4 Department of Cardiology, University of Groningen, University Medical Center Groningen, Groningen, The Netherlands; 5 Department of Critical Care, University of Groningen, University Medical Center Groningen, Groningen, The Netherlands; 6 Department of Surgery - Vascular Surgery, University of Groningen, University Medical Center Groningen, Groningen, The Netherlands; 7 Department of Nephrology, VU University Medical Center, Amsterdam, The Netherlands; 8 Department of Endocrinology, University of Groningen, University Medical Center Groningen, Groningen, The Netherlands

## Correction

After publication of this work [1], we noted that we inadvertently failed to include the complete list of all coauthors. The full list of authors has now been added and the Authors’ contributions and Competing interests section modified accordingly.

## Competing interests

The authors declare that they have no competing interests.

## Authors’ contributions

JvA was responsible for the conception and design of the study, researched data, and wrote the manuscript. HPH contributed to acquisition of data, discussion and reviewed/edited manuscript. MCRFvD contributed to acquisition of data and reviewed/edited manuscript. CPHL and ICCH coordinated inclusion of individuals with CAD and reviewed the manuscript. CJZ coordinated inclusion of individuals with PAD and reviewed the manuscript. MGV contributed to acquisition of data, discussion and reviewed/edited manuscript. BHRW coordinated inclusion of diabetic individuals and reviewed/edited the manuscript. HvG contributed to discussion and reviewed/edited manuscript. JLH was responsible for the conception and design of the study, researched data, contributed to discussion and reviewed/edited manuscript. All authors read and approved the final manuscript.
